# The paradoxical brain: paradoxes impact conflict perspectives through increased neural alignment

**DOI:** 10.1093/cercor/bhae353

**Published:** 2024-09-28

**Authors:** Jonathan Levy, Annika Kluge, Boaz Hameiri, Kaisu Lankinen, Daniel Bar-Tal, Eran Halperin

**Affiliations:** Department of Neuroscience and Biomedical Engineering, Aalto University, Rakentajanaukio 2 C, 02150 Espoo, Finland; Department of Criminology and Gonda Brain Research Center, Bar Ilan University, Max and Anna Webb, 5290002 Ramat-Gan, Israel; Department of Neuroscience and Biomedical Engineering, Aalto University, Rakentajanaukio 2 C, 02150 Espoo, Finland; The Evens Program in Conflict Resolution and Mediation, Tel Aviv University, Chaim Levanon St 55, 6997801 Tel Aviv-Yafo, Israel; Athinoula A. Martinos Center for Biomedical Imaging, Department of Radiology, Massachusetts General Hospital, Charlestown, MA 02129, United States; Harvard Medical School, Boston, MA 02115, United States; School of Education, Tel Aviv University, Chaim Levanon St 55, 6997801 Tel Aviv-Yafo, Israel; Department of Psychology, The Hebrew University of Jerusalem, Mount Scopus, 91905 Jerusalem, Israel

**Keywords:** intergroup interventions, neural alignment, social neuroscience, intergroup conflicts, Meg

## Abstract

Mental perspectives can sometimes be changed by psychological interventions. For instance, when applied in the context of intergroup conflicts, interventions, such as the paradoxical thinking intervention, may unfreeze ingrained negative outgroup attitudes and thereby promote progress toward peacemaking. Yet, at present, the evaluation of interventions’ impact relies almost exclusively on self-reported and behavioral measures that are informative, but are also prone to social desirability and self-presentational biases. In the present study, magnetoencephalography tracked neural alignment, before and after the paradoxical thinking intervention, during the processing of auditory narratives over the Israeli–Palestinian conflict and thereby evaluated the intervention’s potential to change individuals’ (*n* = 80) mental perspectives over the conflict. Compared to baseline, the conflict-targeted intervention yielded a specific significant increased neural alignment in the posterior superior temporal sulcus while processing incongruent as well as congruent political narratives of the conflict. This may be interpreted as a possible change in perspective over the conflict. The results and their interpretations are discussed in view of the critical added value of neuroimaging when assessing interventions to potentially reveal changes in mental perspectives or the way in which they are processed, even in contexts of entrenched resistance to reconsider one’s ideological stance.

## Introduction

For decades, social psychologists investigated the psychological processes underlying formation, preservation, escalation, and de-escalation of intergroup conflicts (Allport 1954; Tajfel and Turner 1979; Mackie et al. 2000; Bar-Tal and Halperin 2011a). In recent years, a new interventionist approach emerged to generate evidence-based knowledge on how to devise and structure interventions that could be useful for practitioners and decision makers for peacefully improving intergroup relations (for a recent review, see Halperin et al. 2023). As such, new studies examined the empirical impact of interventions on the reduction of intergroup prejudice (Paluck 2016; Badea and Sherman 2019) and the moderation of entrenched negative outgroup attitudes (Hameiri et al. 2018; Bruneau et al. 2020; Bar-Tal et al. 2021; Paluck et al. 2021).

Yet, despite the huge number of intergroup interventions and empirical studies assessing them in recent decades, the impact of these interventions remains debated (Paluck 2012; Hameiri et al. 2014a; Paluck et al. 2021). Recently, Paluck and colleagues reviewed 418 experiments applying intergroup interventions, and their quantitative and qualitative assessments suggest a troubling situation: The large majority of intergroup interventions exaggerate effects due to small sample size and publication bias, and find limited effects (Paluck et al. 2021). Given the limited findings about the durable effects of interventions, we need to identify better tools to assess them and to better understand the mechanisms that make them effective. Functional neuroimaging has the potential to do both as it can quantitatively assess cognitive and affective processes, even those that individuals are unwilling to report, are unaware of, or that may trigger future change in behavior and attitudes. Surprisingly, however, out of those hundreds of studies reviewed in Paluck et al. (2021), none has implemented neuroimaging to assess the impact of intergroup interventions, but relied solely on self-reported and behavioral measures that are valuable and informative, but are also limited by social desirability and self-presentational biases (Jost et al. 2014; Bruneau 2015). It is noteworthy that although the past decade has seen an abundance of neuroimaging studies examining the neural underpinnings of intergroup processes (Kubota et al. 2012; Molenberghs 2013; Amodio 2014; Cikara and Van Bavel 2014; Zaki and Cikara 2015; Reynolds and Klik 2016; Scheepers and Derks 2016; Han 2018; Vollberg and Cikara 2018; Klimecki 2019; Amodio and Cikara 2020; De Dreu et al. 2020; Merritt et al. 2021) and conflicts in computerized simulations (Baumgartner et al. 2014; Han et al. 2020; Yang et al. 2020) or in real-life (Levy et al. 2016; Han et al. 2021), neuroimaging was rarely used to study intergroup interventions.

To advance the field of intergroup relations, it is necessary to use the advantages of neuroscience not only to better understand processes but also to radically transform the way that intergroup interventions are assessed and the way their impact is evaluated. In principle, studies in neuroscience succeed in penetrating the mind without any awareness and thus indicate the influence of the intervention. For instance, tracking neural activity was found to be more accurate than traditional self-reported measures in predicting future outcomes in education (Gabrieli et al. 2015), health-related behavior (Falk et al. 2015), and violent behavior (Poldrack et al. 2018). Magnetoencephalography (MEG) is an excellent neuroimaging technique for examining intergroup processes, as it is uniquely predisposed to capturing rhythmic mechanisms and their neural generators (Baillet 2017), and this can allow to examine covert emotions (Levy et al. 2016) and attitudes (Levy et al. 2021) toward intergroup targets. Hence, using neuroimaging to evaluate intergroup interventions can tackle the reported lack of robust impact by intergroup interventions (Paluck et al. 2021) by tapping into the “black box” of the mind.

Examining the mental “black box” is particularly relevant when intricate unconscious barriers and defenses make it very hard to change mental perspectives and attitudes—which can also be defined as “political narratives.” Here, we refer to the latter as “political perspective” similarly to Shenhav's (2006) definition: “*Narrative as Perspective … the very choice of describing certain events and not others establishes a particular viewpoint*” (p. 248). Accordingly, the unfreezing of entrenched narratives is a key characteristic of de-escalation in situations of intractable conflicts between social groups, such as the Israeli–Palestinian conflict (Bar-Tal and Halperin 2011; Levy et al. 2021). These conflicts are widespread across the globe and are characterized by vicious cycles of violence (Bar-Tal and Halperin 2011), as recently instantiated by the October 2023 outbreak of atrocious violence and the unprecedented number of civilian casualties. Because such conflicts intensify ingroup narratives and resistance to peace-making efforts, it increases the number of individuals who fiercely adhere to an ethos (i.e. narratives) of conflict, and it remains a timely challenge to evaluate if certain interventions can change those ingrained conflict perspectives (Bar-Tal 2013; Walton and Wilson 2018).

Several interventions try to counter those conflict narratives by introducing narratives that are nonidentity-threatening in relation to one’s beliefs—for instance, by asking hawkish Jewish-Israelis leading questions that prompt them to respond with answers that are inconsistent (or nonidentity-threatening) with the mainstream narrative among Jewish-Israelis, e.g. “*Why do you think that the Israeli army often acts immorally?*”, but this approach often faces strong resistance (Hameiri et al. 2018). To circumvent the hurdle of reconsidering one’s narratives, the paradoxical thinking intervention was developed. It offers to take these narratives to the extreme, by exaggerating them (Hameiri et al. 2014b; Hameiri et al. 2016; Bar-Tal et al. 2021; Knab et al. 2021), thereby destabilizing individual’s affective state and inducing identity threat (Hameiri et al. 2018). For example, in line with the paradoxical thinking approach, individuals could be asked leading questions that prompt them to respond with answers that are identity-threatening (or consistent with—but much exaggerated than) to the mainstream narratives, e.g. “*Why do you think that it is an absolute fact that the Israeli army is the most moral army in the world, and that this cannot be questioned?*”. Accordingly, such a paradoxical statement challenges the validity of individual’s beliefs and they may feel threat to their identity, feeling affectively disturbed, put off balance, shocked, and surprised. This affective state can result in a change of perspective in a way that would mitigate adherence to the narrative of conflict. Moreover, when operationalized as an interview with a series of typically 10 leading questions, this interventional approach is “neuroimaging-compatible” as it can be promptly evaluated under neuroimaging settings, unlike classic lengthy contact groups that would be very difficult to implement together with neuroimaging.

**Fig. 1:**
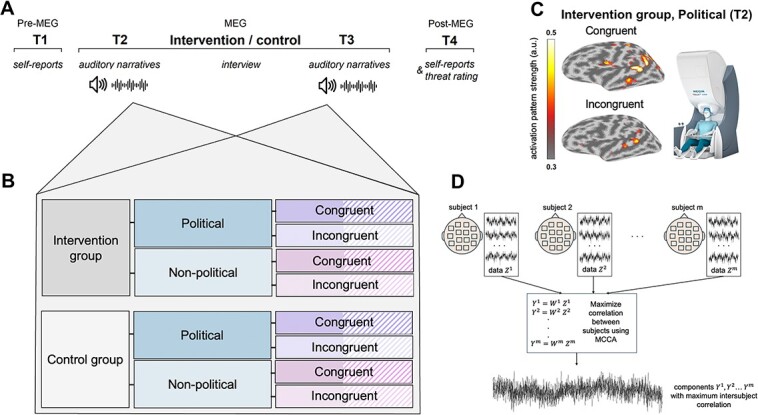
A) Timeline and experimental design from left (T1) to right (T4), reflecting the opening and ending stages of the experiment. The experiment includes a session in MEG (intervention/control, time points T2 and T3), with pre- (time point T1) and post-MEG (time point T4) behavioral sessions. B) During the MEG session, the subjects were presented with audio narratives that were political and nonpolitical. The sentences were different in pre- and postintervention with the following structure: The sentences were the same for both groups, but they were presented in a pseudorandom order for different subjects. Half of these sentences were congruent/incongruent. In addition, we had two sets of the congruent/incongruent sentences for counterbalancing: For half of the sample, a given set of narratives was presented at T2 and the second at T3, whereas for the other half of the sample, it was reversed (i.e. that given set was presented at T3 and the other at T2). In total, there were 32 conditions—16 in each pre/postsession (intervention/control group, political/nonpolitical sentences, congruent/incongruent sentences, two sets of sentences). C) Cortical source activation maps (average across subjects) for the MCCA components while listening, inside the MEG, to congruent (upper) and incongruent (lower) political narratives, preintervention condition (time point T2). Colorbar conveys activation pattern strength of MCCA (arbitrary units). D) Illustration of the principles behind MCCA that was used to extract signal components that correlate maximally across experimental subjects. The method determines weights ${W}^m$ for the neural data ${Z}^m\ of$ each subject so that the resulting projections ${Y}^m$ provide maximal intersubject correlation.

Framed within the Israeli–Palestinian conflict, the current MEG study applied the paradoxical thinking intervention (i.e. exaggeratedly consistent or identity-threatening statements as previously tested (Hameiri et al. 2018) and compared it to a control manipulation, across eighty Jewish-Israelis holding hawkish attitudes regarding the Israeli–Palestinian conflict (data were collected between 2018 and 2019). The a priori preregistered hypothesis (https://osf.io/xj3zb) is that the paradoxical thinking intervention, compared to the control manipulation, would impact and moderate neural processing and self-reported evaluations of narratives about the conflict; in other words, the intervention is thought to distance from the extreme side of the identity/ideological affiliation of individuals as shown in multiple previous behavioral paradoxical thinking studies (Hameiri et al. 2018). As such, the study assessed change in the neural response to Israeli–Palestinian narratives from baseline (T2) to postintervention (T3) and compared this response to the neural response to nonpolitical narratives (Fig. 1). Narratives were presented as naturalistic auditory stimuli (Jääskeläinen et al. 2022). The mental processing of narratives and its neural underpinnings are best captured in neuroimaging using the measure of the neural alignment (e.g. signal similarity) that narratives in movies or in auditory stories evoke across participants in associative and sensorial cortices while processing emotionally arousing stimuli or while intentionally adopting a shared psychological perspective (Hasson et al. 2004; Lahnakoski et al. 2014; Nummenmaa et al. 2018); in MEG signals, this alignment measure can be computed through the multiset canonical correlation analysis (MCCA) (Lankinen et al. 2014, 2018; Levy et al. 2020).

Neural alignment during narrative processing reflects the interpretation of the narratives, as could be shown in a study reporting increased alignment of default mode and fronto-parietal networks among individuals who shared the same interpretation (Nguyen et al. 2019). Critically, even when people perceive identical narratives, if their interpretation changes, this will change the neural alignment during the processing of the narratives; for instance, several studies reported increased alignment of sensorial as well as higher-order cerebral networks (including the default-mode network, language areas, theory-of-mind areas, and subsets of the mirror neuron system), which tended to be similar among people who shared the same interpretation (Yeshurun et al. 2017; Bacha-Trams et al. 2018; Finn et al. 2018). Recent studies with political vignettes report that variations in neural alignment reflect variations in political perspectives, valuations, and attitudes in the prefrontal cortex as well as in limbic (amygdala and striatum), early sensory, motor, somatosensory, and mentalizing regions (Leong et al. 2020; Van Baar et al. 2021; De Bruin et al. 2023; Katabi et al. 2023) and can predict future change in political attitudes (Leong et al. 2020). Accordingly, an increase in neural alignment while processing auditory narratives specific about the conflict following the intervention would reflect a change in the perspective (Bacha-Trams et al. 2018, 2020) and the interpretation (Bacha-Trams et al. 2017; Leong et al. 2020; Komulainen et al. 2021; Katabi et al. 2023) of narratives about the conflict. Such change in perspective is the target of the intervention as it can mitigate fierce psychological resistance to peace-making efforts.

## Materials and methods

### Experimental subjects

#### Intervention

Eighty Jewish-Israeli adults were recruited to this preregistered (https://osf.io/xj3zb) randomized controlled trial (RCT) study through advertisement at Bar-Ilan University. The precise final sample size was preregistered as it was considered a large sample for MEG studies (Chaumon et al. 2021); previous MEG intervention (Hautala et al. 2022; Levy et al. 2022) and MEG MCCA (Lankinen et al. 2014, 2016, 2018) studies found between-subject effects with much smaller samples. Based on this sample, a sensitivity power analysis (Faul et al. 2007) indicated that our analysis was sufficiently powered to detect a medium effect size (Cohen’s *d* = 0.47) with 80% power of the within subject change across time in the paradoxical thinking condition.

The study received approval from the Reichman University ethics committee, and participants gave written informed consent before the study in line with the university’s Institutional Review Board. Participants received monetary compensation for their participation and were informed that they can leave at any point or drop out of the study. They were prescreened for no serious medical, neurological, or psychiatric conditions and for MEG metal compatibility (body metal-free, including tooth bracelets, implants, and piercing).

In this RCT design, we conducted a direct replication to our previous study (Hameiri et al. 2018), but this time inside the MEG. Accordingly, participants were randomly assigned to either undergo the paradoxical-thinking intervention (Hameiri et al. 2014b) or a control (i.e. inconsistency) manipulation—both in the form of interview (Hameiri et al. 2018) using 10 open questions (see Supplementary material). The aim of the intervention was to moderate hawkish attitudes related to the Israeli–Palestinian conflict among Jewish-Israeli individuals who tend to oppose peace-making policies (Bar-Tal et al. 2012). The intervention followed the paradoxical thinking principle: interviewing participants (audio-visual interview, while the interviewer and interviewee watched each other on a video screen) while raising questions that are in line with their political ideology but extreme, exaggerated, or even absurd (Hameiri et al. 2014b) (e.g. “Why do you think that it is an absolute fact that the Israeli army is the most moral army in the world, and that this cannot be doubted?”). By contrast, the control condition follows the inconsistency principle: interviewing participants while raising questions that are totally identity-threatening in relation to their political ideology (e.g. “Why do you think that the Israeli army often acts immorally?”). The control condition is not expected to lead to any moderation of the targeted ideological attitudes (Hameiri et al. 2018).

Noteworthy, data from one participant were discarded as a technical error resulted in partial data recording. Hence, the final dataset for analysis included 79 individuals: 37 (41% females) assigned to the intervention and 42 (50% females) assigned to the control condition.

#### Demographic and ideological variables

Table S1 details the demographic and political parameters in the sample. Subjects were young (24.68 ± 3.72 years) participants with a level of education on average between a professional certificate and a bachelor university degree, and most of them (72.36%) are residing in the extended Tel-Aviv Metropolitan area, thereby providing a relatively good representation of the Israeli population with a slight bias to that Metropolitan area. As detailed on Table S1, on average, their education level was between studying for a certificate and bachelor level, and the religiosity on average leaned toward “traditional,” which in Israel is considered a light form of Jewish religiosity. Adherence to the ethos of conflict in Israel captures conflict-related ideology in Israeli and was found to be negatively associated with support for peace-making policies (Bar-Tal et al. 2012). This is why we used this scale to prescreen the participants and include Israeli-Jewish individuals who tend to oppose peace-making policies with the Palestinians (i.e. an ethos score equal or superior to three was used as threshold for opposing peace agreement; 1—highly endorse, 3—slightly oppose, 6—highly oppose) (Hameiri et al. 2018). As expected, Table S1 shows that participants in the study tended to strongly support the Israeli ethos. Additionally, self-reported ideology was on average moderate right-wing. Hence, the subjects in this study had right-wing ideology and relatively high support of the Israeli ethos, without significant difference between the intervention and control groups.

### Experimental design

In this intervention study, the aim was to evaluate the potential interventional impact on neural and self-reported measures of narratives/attitudes of the conflict. To this end, before and after the intervention (c.f. above: the intervention subsection), we used exposure to narratives as well as self-reported attitudes (Fig. 1A and B). During the exposure to narratives, we measured neural alignment underlying political and nonpolitical narratives, and the self-reported evaluations of those narratives. The pre- and postsessions lasted approximately 10 min each, including the rating time required for each statement. In addition, outside the MEG, participants reported their attitudes before and after the MEG experiment (T1 and T4).

#### Narratives (inside the MEG)

Narratives were generated based on local media editorials, letters to the editor, blogs, and websites similarly to a previous study (Bruneau and Saxe 2010) and were piloted among 140 right-wing Israelis to validate that congruent and incongruent narratives indeed were rated inversely to each other on *Truthness* and that political narratives induced more *Affect* than nonpolitical narratives (see Supplementary material). Two narrative conditions were used to evaluate attitude moderation, politically congruent (e.g. “*The situation will not improve until the Palestinians stop inciting terrorism and hatred, and accept the right of Israelis to live freely in their country. Israel has already accepted the two-state solution in the past; it is the Palestinians who did not accept it.*”) and incongruent (e.g. “*Instead of ignoring repeated demands from most countries of the world, including the USA, Israel must stop building and expanding settlements in the territories of the Palestinian Authority.*”)—from an Israeli right-wing ingroup perspective (for a detailed text list of narratives, see Supplementary material). The narratives related to the Israeli–Palestinian conflict, as was used in a similar way in previous research (Bruneau and Saxe 2010). In addition, we included two control nonpolitical narratives: congruent (e.g. “*Wind turbines generate electricity from wind energy and thereby help reduce air pollution and greenhouse gases; Therefore, their use to generate electricity has been adopted in some countries of the world..*”) and incongruent (e.g. “*The claim that there is a sharp increase in the average temperature of the earth’s surface during the last hundred years must be doubted, since those who claim so are scientists who falsified temperature data.*”).

The narratives were expressed as auditory statements that were prerecorded by a professional radio personality. They were rated before and after the intervention/control inside the MEG on perceived *Truthness* of the narratives and the *Affect* that they aroused. In total, each participant perceived 48 narratives, 12 from each of the four narrative types, half of which were in the before and the other half at the after intervention/control session.

### MEG narrative data acquisition and analysis

#### MEG recordings

We recorded ongoing brain activity (sampling rate, 1,017 Hz, online 1–400 Hz band-pass filter) using a whole-head 248-channel magnetometer array (4-D Neuroimaging, Magnes 3600 WH) inside a magnetically shielded room in the Gonda Brain Center at Bar-Ilan University. Reference coils located approximately 30 cm above the head, oriented by the *x*, *y*, and *z* axes, enabled removal of environmental noise. Movements were visually monitored by the experimenter via a camera and by a movement-tracking system using five coils attached to the participants’ scalp to record head position relative to the sensor array.

#### MEG data preprocessing

To preprocess MEG and extract from it the relevant data for our planned analyses, external noise (e.g. powerline, heartbeat, and mechanical vibrations) was first reduced automatically using a predesigned algorithm (Tal and Abeles 2013). Next, the data were visually inspected, and noisy channels and data segments were removed. The data were filtered between 0.2 and 400 Hz and resampled at 400 Hz. Thereafter, independent component analysis (Hyvärinen 1999, ICA, FastICA algorithm) was applied to decompose the data into 30 ICA components. The ICA components responsible for electro-oculography (EOG) artifacts were visually identified from the ICA topographies and ICA time-series and removed from the data. As the MCCA requires that the length of the MEG data are the same for each subject, the time instances of the rejected time segments were padded with zeros. After zero-padding, the data were filtered into 0.5 to 10 Hz band (a zero-phase forward-backward filter, transition band 0.5 Hz). The data were preprocessed with MATLAB 2019b and FieldTrip toolbox (Oostenveld et al. 2011).

#### MEG spatial filtering with MCCA

To examine neural alignment of MEG data, we used a multivariate spatial-filtering approach based on MCCA (Kettenring 1971; Li et al. 2009) to uncover consistent brain signals across subjects (Lankinen et al. 2014). With this approach, we can find the maximal correlation between the subjects’ sensor-space signals in a data-driven manner, without assumptions about the location of the sensors relative to the anatomy. This is a major advantage compared to univariate sensor-to-sensor or corresponding source time course correlations where assumptions of functional anatomy and sensor locations are tighter.

In the multiset canonical correlation approach, the weight vectors for each dataset (here weights for sensors of each subject) are determined such that the resulting canonical variates achieve maximum overall correlation between the datasets. Spatial filtering refers to projection ${\mathbf{Y}}^m={\mathbf{W}}^m{\mathbf{Z}}^m$, where the output ${\mathbf{Y}}^m$is a weighted sum of the multidimensional signal ${\mathbf{Z}}^m$($D\times t$ matrix, where $D$ is the dimension of the signals, and $t$ is the number of time points). Here, the superscript $m$ refers to the dataset of one subject ($m=1\dots M$, where $M$ is the number of subjects). The resulting projections in rows of ${\mathbf{Y}}^m$($D\times t$ matrix), i.e. the canonical variates, are mutually uncorrelated but maximally correlated between the subjects. Here, spatial filter weights ${\mathbf{W}}^m$ in MCCA were calculated by using the MAXVAR cost function (Kettenring 1971). We used PCA to reduce the dimensionality of the sensor-level data to 50 (capturing approximately 98.7% of the variance in the data) before applying MCCA.

We used a 3-fold cross-validation for model training and testing. More specifically, the 1.5-min data were divided into three nonoverlapping parts, and the MCCA model was trained three times using a different part as a test data and the rest of the data as training data. The MCCA model was trained and the data were analyzed separately and identically for all conditions, sessions pre and post, and for the two groups (control and intervention). Additionally, because there were two versions of stimuli that were pseudorandomly assigned across participants, we analyzed separately per version, therefore maintaining within-group correlation of participants exposed to identical stimulation. The estimated spatial filter weights were applied to each corresponding test set, and only the resulting concatenated test data (canonical variates) were used in further analysis.

For visualization, the weights ${\mathbf{W}}^m$ ($D\times D$, $D$ is the number of PCA components) were transformed back to the dimensions 248 × 248 corresponding to the number of original MEG channels. To better enable physiological interpretation of the spatial filter weights, we converted the spatial filter weights to activation patterns (forward models) (Haufe et al. 2014). This procedure refers to finding the activation pattern$A={\Sigma}_z{\mathbf{W}}^{\prime m}{\Sigma}_y$, where ${\Sigma}_z$and ${\Sigma}_y$are the covariance matrices of the data ${\mathbf{Z}}^m$and projections ${\mathbf{Y}}^m$.

#### Correlation analyses and statistical testing

Neural alignment during narrative processing reflects the interpretation of the narratives (Nguyen et al. 2019), and an increase in neural alignment reflects a change in the perspective (Bacha-Trams et al. 2018, 2020) and the interpretation (Bacha-Trams et al. 2017; Leong et al. 2020; Komulainen et al. 2021; Katabi et al. 2023) of narratives.

To compute neural alignment during the processing of narratives of the conflict, MCCA was calculated separately for the intervention and control groups, for the two versions within each group, for the two experimental sessions (pre and post), and for the experimental conditions within each group (i.e. narratives). After running MCCA for each of the sets above, we calculated pairwise Pearson’s correlations between the subjects within each test set. Next, we applied Fisher’s *z*-transformation to the correlation coefficients $r$ before calculating an average correlation $\overline{z}$ (MCCA intersubject correlation) over all the pairwise combinations. Statistically significant *z*-values were transformed back to correlation coefficients by Fisher’s inverse *z*-transformation. For further analysis, we picked only the MCCA component in each test set that had the strongest correlation between the subjects. In order to examine neural alignment that is significant, we tested the statistical significance of the selected MCCA components by nonparametric circular bootstrapping (Fig. 1D). The null distribution was estimated by circular shifting each subjects’ time series 5,000 times with random lags and then calculating an average of the correlations of the shuffled subject pairs. The 3 cross-validation sets were then combined by calculating an average of absolute values of 3 cross-validation sets, both for original correlation values and null distributions. ﻿The *P*-values were estimated using these averaged correlation values. The significance threshold was set at *P* < 0.05, corrected for multiple comparisons between groups and conditions by false discovery rate (FDR) procedure (Benjamini and Hochberg 1995). To address the hypothesis that neural alignment is modulated by the intervention vs control, we compared MCCA values for each of the narratives’ conditions (incongruent and congruent) and its nonpolitical controls, separately for the intervention and control groups. The groups and conditions were compared with a independent-samples *t*-test.

#### Cortical source analysis

To further pinpoint the mental functions that neural alignment captures, we examined the cortical regions from which the alignment emerges. To this end, the sensor-level activation patterns were transformed to the anatomical source space by employing minimum-norm estimates (Hämäläinen et al. 1993) with MNE-python (Gramfort et al. 2013). Thus, for one MCCA component, the input for MNE was a 1 by 248 vector. Individual activation maps of the canonical components of interest were projected onto individual source spaces calculated using the individual MEG data and a common template (“fsaverage” in FreeSurfer—http://surfer.nmr.mgh.harvard.edu/). The source activations were then averaged across participants.

### Self-reported attitudes (outside the MEG)

In addition to the self-reported rating of the narratives inside the MEG, participants also reported attitudes outside the MEG, before (T1) and after (T4) intervention (Fig. 1), as a replication to our previous behavioral study (Hameiri et al. 2018) that used the following measures: (i) *Ethos*, (ii) *Openness,* and (iii) *Moral conviction*. They also evaluated two measures that were sampled only after the intervention: *Unfreezing* and *Support for peace-supporting policies* (Hameiri et al. 2014b; Hameiri et al. 2016).

### Rating of identity threat and affective disturbance during the intervention (outside the MEG)

Finally, it has been suggested that the paradoxical intervention operates by destabilizing an individual’s affective state and in particular by inducing identity threat, affective surprise, disturbance, and confusion (Hameiri et al. 2018). Hence, after the MEG session, at T4, we used a questionnaire to examine the (i) *Affective Disturbance* experienced during the intervention (9 items, e.g. “I was not at ease during the interview.”). We additionally video-recorded the interviews, and then, after the MEG session, we showed to each participating individual the video of the interview s/he went through. We explained what identity threat is and asked them to continuously rate (from 1 to 10), via a designated homemade software, the level of (ii) *Identity Threat* that they experienced at each moment of the interview, similarly to previous research (Raz et al. 2012). The aim of this procedure was to evaluate the maximal level of identity threat that the intervention may have triggered.

## Results

### The effect of the intervention on neural alignment underlying political and nonpolitical narratives

Before addressing the interventional hypothesis, an initial examination of the MCCA patterns focused on the spatial localization of these patterns at the T2 session, that is, before any intervention/control, and for the full sample. Furthermore, source localization for the strongest activation of the significant MCCA component revealed superior temporal gyrus activation for both types of political narratives (Fig. 1C, lateral surface maps). Interestingly, incongruent narratives additionally activated the dorso-medial prefrontal cortex (dmPFC). These results suggest that the processing political narratives activated social cognition of auditory information for the interpretation of narratives, in line with previous studies (Beauchamp 2015; Yeshurun et al. 2017; Finn et al. 2018; Nguyen et al. 2019; Katabi et al. 2023). In particular, recent research highlighted the role of the dmPFC in the emotional–moral processing of political narrative content (Leong et al. 2020), thereby explaining its involvement in the processing of incongruent narratives in the present data at T2.

**Fig. 2:**
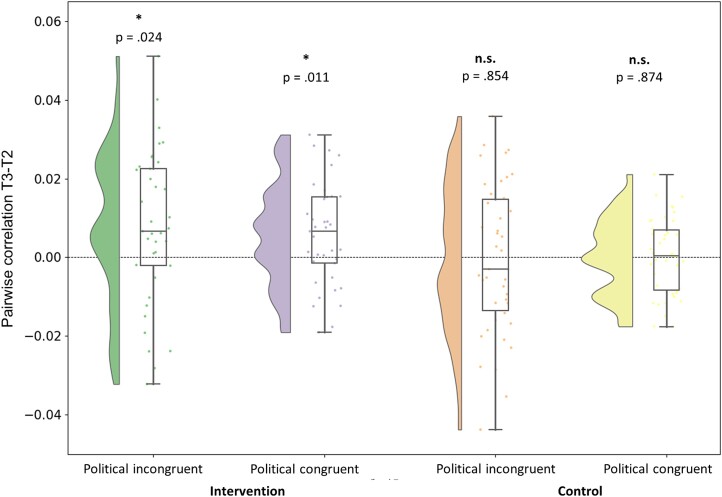
Political narratives. The difference in T3-T2 MCCA correlation values for the component with the strongest correlation in the test set, groupwise. The violin plots illustrate the distribution of correlation value differences participant-wise. The box plots are drawn from the first to the third quartile of individual correlation value differences. The line in the box plot is the median value. The dots represent individual values, and the error bars are at 95% confidence intervals.

**Fig. 3:**
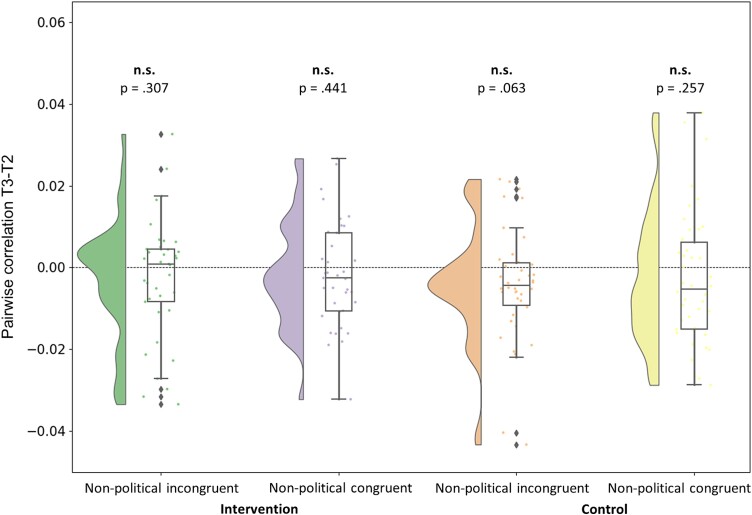
Nonpolitical narratives. The difference in T3–T2 MCCA correlation values for the component with the strongest correlation in the test set, groupwise. The violin plots illustrate the distribution of correlation value differences participant-wise. The box plots are drawn from the first to the third quartile of individual correlation value differences. The line in the box plot is the median value. The dots represent individual values, and the error bars are at 95% confidence intervals.

Then, to address the preregistered hypotheses that the intervention, not the control manipulation, would moderate the political attitudes (i.e. further distancing from the extreme side of the identity/ideological affiliation of individuals) first *at the neural level*, we examined political attitudes as evaluated via the MCCA analysis (i.e. neural alignment) before and after the intervention, as well as their functional mapping as reflected via source localization. It is noteworthy that the MCCA values for different groups come from different MCCA models and thus cannot be assumed to yield fully comparable values between those the groups. Thus, we focused on the within-group level analysis in the current study, demonstrating pre/postintervention effects on the types of processed sentences. Statistically significant differences between the session before (T2) and after (T3) the intervention were detected (Fig. 2); MCCA values significantly increased after the intervention in both politically incongruent [*t*(36) = 2.362, *P* = 0.024, Cohen’s *d* = 0.388] and congruent [*t*(36) = 2.669, *P* = 0.011, Cohen’s *d* = 0.439] conditions. By contrast, there was no significant difference in neither incongruent [*t*(41) = −0.185, *P* = 0.854, Cohen’s *d* = −0.029] nor congruent [*t*(41) = 0.160, *P* = 0.874, Cohen’s *d* = 0.025] conditions in the control group. These results suggest that the intervention increased neural alignment across subjects while processing the political narratives. This increase is often interpreted as enhanced similarity in sensory processing (Katabi et al. 2023) or even in higher-order cognitive perspectives (Lahnakoski et al. 2014) and can therefore point out an intervention-driven increase in similarity among individuals in the way they process political narratives. Furthermore, Fig. 3 illustrates no significant neural alignment effects on the nonpolitical narratives: intervention nonpolitical incongruent [*t*(36) = −1.035, *P* = 0.307, Cohen’s *d* = −0.170; intervention nonpolitical congruent [*t*(36) = −0.779, *P* = 0.441, Cohen’s *d* = −0.128; control nonpolitical incongruent [*t*(41) = −1.916, *P* = 0.062, Cohen’s *d* = −0.296; control nonpolitical congruent [*t*(41) = −1.149, *P* = 0.257, Cohen’s *d* = −0.177]. Hence, these results suggest not only that the intervention increased the neural alignment across subjects while perceiving the political narratives but also that this increase targeted well the relevant political attitudes rather than a general nonspecific effect.

Moreover, at the cortical source level, we found that the effect of the intervention on both types of political narratives peaked in the posterior superior temporal (see Fig. S1, left panels). This suggests that the interventional effect impacted the same narrative-specific regions that were revealed in the full sample before the intervention—by increasing their alignment across participants. By contrast, in the control group, the (insignificant) effect of the control manipulation peaked slightly more posteriorly: in the parietal (precuneus) and posterior superior temporal regions (see Fig. S1, right panels), yet, it would be precautious to draw conclusive interpretations based on these localizations, particularly as they were not statistically significant. To further investigate the specificity of those interventional effects on political narratives, we contrasted them to the interventional (insignificant) effects on nonpolitical narratives. The resulting sources remained basically unchanged: in the posterior superior temporal cortex (see Fig. S2). It is noteworthy that although it is more robust to rely on the sensor data given the lack of individual MRI scans for each participant, overall, the source projections from the multiple MCCA contrasts consistently consolidate the sensor-level findings pointing out the impact of the intervention on the alignment of activity in narrative-specific areas of the brain.

### The effect of the intervention on self-reported evaluations of and affective reactions to political and nonpolitical narratives

Second, to address the preregistered hypotheses that the intervention, not the control manipulation, would moderate the political attitudes at the self-reported level, we examined the self-reported rating of perceived *Truthness* of the narratives and the *Affect* that they aroused—before and after the intervention. The various narratives were rated (see Supplementary material) as expected on *Truthness* (i.e. low and high in the incongruent and congruent conditions, respectively) and on *Affect* (i.e. medium and low in the political and nonpolitical conditions, respectively), in line with the pilot data (SI), and the intervention did not yield any significant effect on the self-reported ratings of these two variables (SI). Similarly, the intervention did not yield any significant change in the self-reported constructs of *Ethos*, *Openness*, *Moral conviction, Unfreezing*, and *Support for peace-promoting policies* (SI)*.*

Furthermore, we examined the subjective experience of *Affective Disturbance* and *Identity Threat* during the intervention. *Affective Disturbance* was not significantly [*t*(76) = 0.96, *P* = 0.34] different in the intervention (2.56 ± 0.70/6) compared to the control (2.73 ± 0.83/6) group. Yet, *Identity Threat* was almost significantly [*t*(64.28) = −2.00, *P* = 0.050] higher (Cohen’s *d* = −0.48) in the intervention (7.34 ± 2.32/10) compared to the control (6.03 ± 3.09/10) group. Interestingly, the two measures of *Affective Disturbance* and *Identity Threat* were significantly positively correlated (*r* = 0.39, *P* = 0.001) with each other. These results suggest that while the measures of *Affective Disturbance* and *Identity Threat* are highly related to each other, only the latter is significantly elevated by the paradoxical intervention compared to the control condition. This result consolidates previous studies emphasizing the elevation of *Identity Threat* as a psychological process enabling the impact of the paradoxical thinking intervention (Hameiri et al. 2018).

Finally, to examine whether the neural effects of the intervention may be explained by the self-reported measures above (i.e. *Truthness*, *Affect*, *Ethos*, *Openness*, *Moral conviction, Affective Disturbance*, and *Identity Threat*), a correlation was computed; however, none of those measures could significantly explain the neural effects neither in the politically incongruent (*P_un-corrected_* > 0.11) nor in the politically congruent (*P_un-corrected_* > 0.10) conditions. This suggests that the neural effects of the intervention were orthogonal to all other behavioral and self-reported measures.

## Discussion

The turn of 2023 in the Middle East was characterized by brutal violence and an unprecedented number of civilian casualties among Israelis and Palestinians. Such cycles of violence intensify ingroup narratives and resistance to peace-making efforts (Bar-Tal and Halperin 2011), and therefore, a pressing challenge remains to determine whether certain interventions can change ingrained perspectives (i.e. narratives) over conflicts (Walton and Wilson 2018). In order to investigate the effect of intervention on a change of conflict supporting attitudes, the present study implemented the paradoxical thinking intervention vs control condition, as Israeli–Jewish individuals with hawkish perspectives on the conflict underwent MEG while perceiving narratives about the Israeli–Palestinian conflict. The a priori preregistered hypothesis of this study was that the paradoxical thinking intervention, compared to the control manipulation, would moderate the political attitudes, as evaluated by the neural alignment and self-reported measures. The results showcase a clear pre- vs postintervention impact at the neural level, no impact at the self-reported level, and a certain impact at the self-reported experience during the intervention. This is in line with the results from a recent MEG study evaluating implicit neural bias (Hautala et al. 2022), but here, we additionally show, in a large sample for a MEG study, that the intervention can impact directly the processing of narratives. This suggests that the intervention impacts the mind of individuals in two dimensions: at the unconscious/implicit level as reflected by neural activity modulation and at the conscious/explicit level as reflected by self-reports; the latter may be less sensitive to detection than the first. This is also in line with other recent studies that examined changes in cognitive and affective processes in the political climate of Israel and have not found self-reported differences but revealed significant differences in their neural underpinnings (Levy et al. 2016, 2021, 2022; Zebarjadi et al. 2023; Kluge et al. 2024a).

Hence, following what was recently proposed, that the impact of current intergroup interventions is limited (Paluck et al. 2021), the present findings suggest that some interventions, such as the paradoxical thinking, may significantly impact the “black box” in the mind and subsequently change the way that individuals process narratives about the intergroup conflict they adhere to. This is done by penetrating the mind unobtrusively and monitoring the covert influence of the intervention. Thus, the present study advances the field of intergroup relations while highlighting the importance of using neuroimaging in the design and evaluation of intergroup interventions, in particular with interventions such as the present one, which can well suit neuroimaging settings. This is acutely timely and societally relevant in situations of intractable conflicts that are characterized by intricate unconscious barriers, defenses (Bar-Tal and Halperin 2011), and neural control mechanisms (Levy et al. 2021)—thereby resulting in extreme suffering, violence, and causalities.

The processing of political (both incongruent and congruent) narratives has involved mainly the neural alignment of the superior-/middle-temporal sulcus (STS/MTS), and additionally the dmPFC for politically incongruent narratives. The STS and in particular its posterior part (pSTS) is a hub of social cognition (Beauchamp 2015; Levy et al. 2017), social language perception, and mentalizing (Deen et al. 2015; Yang et al. 2015), and previous research has reported the neural alignment of these regions during the processing of social (Finn et al. 2018) and political narratives (Leong et al. 2020; Katabi et al. 2023). Functionally speaking, the neural alignment in the activation of these regions during the processing of political narratives is likely to reflect the interpretation of the narratives (Yeshurun et al. 2017; Nguyen et al. 2019). In this study, we found that this neural alignment significantly increased following the intervention, not the control manipulation, and selectively for the political narratives (and not for the nonpolitical control narratives). The functional interpretation of this increased alignment selectively for the political narratives suggests that individuals undergoing the intervention may have changed their perspective and interpretation of the political narratives. Interestingly, the processing of political narratives changed whether it was congruent or incongruent to participants’ political views. In other words, for individuals in the context of intractable conflicts, the existing narrative is often ideologically hawkish, so the possible change in perspective implies that they reconsider the way they process political narratives—those they previously tended to agree, or disagree with, to a certain extent (Oren et al. 2015).

Past studies showed that neural alignment of cortical regions including in the STS/MTS and the dmPFC can explain the change of perspective (Bacha-Trams et al. 2018, 2020) and the interpretation (Bacha-Trams et al. 2017; Leong et al. 2020; Komulainen et al. 2021; Katabi et al. 2023) of social, political, and affective narratives. Furthermore, such increased neural alignment may reflect learning of new information (Dikker et al. 2017; Meshulam et al. 2021) specifically in the STS And dmPFC. Two recent studies suggest that neural alignment in particular in the dmPFC reflects the processing of political information that is sensitive to individuals’ ideological views and can even predict change in political attitudes. Leong and colleagues reported that neural alignment in the dmPFC intensified during the perception of videos that included risk-related and moral–emotional language, highlighting content features most likely to drive divergent interpretations between politically hawkish and dovish individuals (Leong et al. 2020). That study additionally found that neural alignment in this region predicted change in ideological attitudes in the direction of that group’s position. Another study by Cohen et al. (2024) reported that neural alignment in the dmPFC predicted persuasiveness of political video propaganda. It is therefore likely that the increase in neural alignment in this region following the intervention reflects a change in the individual’s perspective and interpretation of the political narratives that were applied.

Yet, one should consider other interpretations for the interventional increase in neural alignment. For instance, it could also mean that the intervention made political narratives to be perceived more similarly or processed with increased vigilance across the intervened individuals, which may occur if individuals’ group identity was threatened by the paradoxical thinking intervention as was shown by the threat reports and previous paradoxical thinking studies (Hameiri et al. 2018). It is additionally noteworthy that: (i) the increased alignment is less likely to have resulted from perceptual compared to conceptual processes, as the stimuli were not identical across sessions, but pseudorandomly shuffled; furthermore, (ii) the interpretation of perspective change is rather plausible in view of multiple past behavioral studies in various contexts showing that the paradoxical thinking intervention consistently moderates intergroup attitudes (Bar-Tal et al. 2021; Hameiri et al. 2014b; Hameiri et al. 2016, 2018; Knab et al. 2021) and, even recently, that its neural underpinnings may predict future affect change toward outgroups (Kluge et al. 2024b).

The intervention did not only impact neural alignment during the processing of political narratives, but it also increased the experience of threat to one’s identity, as evaluated by the participants watching themselves in a video, which was taken during the intervention inside the MEG room. This self-evaluation took place outside the MEG room and after the completion of the experiment, which allowed for a more detached third-person evaluation of one’s internal experience during the intervention. This approach is in line with previous empirical (Raz et al. 2012; Zebarjadi et al. 2021) and theoretical (Levy and Bader 2020; Jääskeläinen et al. 2022) works examining the interrelationships between inner experiences and neural processes and them providing a good reflection of the underlying psychological underpinnings. In another recent study examining the effect of an intergroup intervention on attitudes between Israelis and Palestinians, in addition to modulating the neural mechanisms of attitudes, it also revealed an interventional effect on attitudes that were sampled by an in-depth lengthy interview; by contrast, no significant effects were found on any other self-reported attitudes and affect (Levy et al. 2022). Hence, it may be that not only neural measures but also in-depth behavioral evaluations may be more sensitive to interventional effects compared to more traditional and brief self-reported measures.

The findings here provide an evidence-based perspective on the relationship between the subjective experience of threat during the intervention and its consequences on the interpretation of narratives. This adds important empirical support to the theoretical assumption that the paradoxical intervention operates by inducing threat to self-identity as antecedence to the modulation of intergroup attitudes (Hameiri et al. 2018). Noteworthy, the modality of the intervention does not seem to matter much as previous studies (Hameiri et al. 2018; Levy et al. 2022; Kluge et al. 2024b) showed that various forms and modalities (visual, audio, audio-visual, reciprocal audio-visual interview) of implementing the paradoxical thinking interventions yield and impact on individuals.

One outstanding question is whether the impact at the neural level and not at the self-reported level reflects anything about the impact of the intervention—does it mean that the effect is subtle, unconscious, and covert? If so, would that mitigate the importance of the impact or, in turn, highlight effects that are hidden or unaware by individuals? Relatedly, the intervention yielded an effect on neural alignment and identity threat, but the effects were not correlated with each other. This pattern is in congruence with previous intergroup intervention and neural alignment studies that did not report any relationship between the neural and behavioral effects (Hautala et al. 2022; Levy et al. 2022), including studies measuring neural alignment of social processes (Bacha-Trams et al. 2017, 2020). Future studies are needed to further examine whether neural and behavioral evaluations convey orthogonal operating channels, in particular in the contexts of intergroup interventions. Furthermore, building on the approach we used in the current research, we propose here another avenue of future investigation: harnessing neuroscience to improve the interventions themselves by recording the neural mechanisms during the course of intervention and a moment-by-moment monitoring of the way that each interventional stimulus may alter neural activity underlying mental processes. A recent study in another societal context found that the neural underpinnings of the paradoxical thinking intervention may predict relatively long-term (self-reported) affect change toward outgroups (Kluge et al. 2024b). More research is needed in these new horizons of exploration to pinpoint specific contents within the intervention that destabilize and unfreeze individual minds and predict long-term impact in behavioral and attitudes.

## Conclusion

The present study advances the field of intergroup relations and showcases a novel strategy of implementing neuroimaging to study the impact of selected intergroup interventions. It reveals that the paradoxical thinking intervention can impact the way that narratives about the intergroup conflict are processed in the mind while at the same time increasing the sense of threat to self-identity. The strategy of paradoxical thinking can therefore be of much value in changing ingrained negative outgroup narratives that, as a corollary, unfreezes resistance to peace-making efforts. In addition to the timely societal value in de-escalating violent conflicts, the results reported here underscore the critical added value of neuroimaging when assessing interventions to reveal a potential change in mental perspectives or at least the way they are being processed.

## Supplementary Material

ms_mcca_SI_revised_bhae353

## Data Availability

Data can be shared upon reasonable request pending institutional approval. Code can be shared upon reasonable request. The study will be under an open access license.
