# Supplementary material for: The paradoxical brain: paradoxes impact conflict perspectives through increased neural alignment
Source: Cereb Cortex. 2024 Sep 28;34(9):bhae353. doi: 10.1093/cercor/bhae353 (PMC11439920; doi:10.1093/cercor/bhae353)
Supplement: ms_mcca_SI_revised_bhae353 [file ms_mcca_si_revised_bhae353.docx]

# **Supplemental Information**

# **The Paradoxical Brain: Paradoxes Impact Conflict Perspectives through Increased Neural Alignment**

# **Methods**

**Subjects.** Participants had a relatively high tendency for ethnocentricity. Right-wing authoritarianism (RWA) score was on average between supporting and opposing RWA but leaning to slightly opposing RWA values.

**Table S1**. Demographic and ideological variables. The level of education was evaluated with the following discrete variables: 1-high-school graduate, 2-professional certificate, 3-Bachelor level, 4-Master’s level and above. Their level of religiosity was evaluated with the following discrete variables: 1-Atheist, 2-Secular, 3-Secular/Traditional, 4-Traditional, 5- Religious 6-Ultra-orthodox. Ideology spanned from 1-extreme right, 2-right, 3-moderate right, 4-center, 5-moderate left, 6-left to 7-extreme left.

| **Demographics** | *M ± SD* | *Between-group p-value* | *Between-group t-value* |  |
| --- | --- | --- | --- | --- |
| Age | 24.68 ± 3.72 | .84 | (1,74) =.20 |  |
| Education | 2.43 ± 1.04 / 4 | .48 | (1,74) = -.71 |  |
| Religiosity | 3.46 ± 1.34 / 6 | .57 | (1,74) = -.57 |  |
| **Political variables** |  |  |  |  |
| Ethos | 4.59 ± .73 / 6 | .22 | (1,74) = -1.24 |  |
| Ideology | 2.99 ± .99 / 7 | .13 | (1,74) = 1.52 |  |
| RWA | 3.15 ± .67 / 6 | .92 | (1,74) = -.10 |  |
| Ethnocentricity | 4.30 ± 1.23 / 6 | .36 | (1,74) = -.92 |  |

**Validation of the narratives – Pilot study.** Although similar narratives were used in a previous study (76), we conducted a pilot consisting of two online surveys sampling 140 right-wing Israelis. In the first survey, anonymous volunteers were asked to judge how clear the narratives are, and in a second survey, they were asked to rate how “true” and how much “affect” the narratives evoke. Results from the first survey indicated that narratives were on average very clear 4.76/5 without statistically significant difference (p > .4) in clarity among the four statement conditions. Results from the second survey indicated that congruent narratives (whether political or non-political) were on average rated as “true” (4.20/5 and 4.09/5) without statistically significant difference (p > .25) between the two. By contrast, incongruent narratives (whether political or non-political) were on average rated as untrue (1.81/5 and 1.66/5) without statistically significant difference (p > .2) between the two. In terms of “affect”, political narratives were on average more arousing than control narratives (p < .000001). These results validated the four conditions.

**Design of the narratives.** Narratives were on average 25.37 words long without statistically significant difference (p > .2) in length among conditions. They were delivered as auditory via the E-Prime® software (Psychology Software Tools Incorporated) in a counterbalanced order, to avoid unspecific stimulus or structure effects. In total, 48 auditory narratives were used before (24) and after (24) the intervention – 6 narratives from each condition, in each session (pre and post intervention). To avoid any confound related to the stimuli themselves, we created two versions of stimuli. Half of the participants perceived the first version in the pre session and the second version in the post session, whereas this was reversed in the second pool of participants.

**The Questions used in the Intervention and control interviews (translated from Hebrew)**

1. Paradoxical thinking (identity-threatening) intervention

A short while ago you were asked to answer a short questionnaire that examined attitudes regarding the Israeli-Palestinian conflict. The aim of this part of the experiment is to examine these attitudes more deeply with a short interview. I ask you to think thoroughly about the questions I am about to ask you, and also to provide the reasoning for your answers. Let's begin:

1. Why do you think that the Palestinians were not victimized by us at all during the conflict’s history, and they do not have the right to claim that they were victimized through the years?

2. Why do you think that us, the Jewish people, are morally superior to all Arabs, so much so that even the most corrupt Israeli criminal is more moral than the most merciful Palestinian doctor?

3. Why do you think that the real and only goal the Palestinians have in mind is to annihilate us, in a manner that transcends their basic needs such as food and health?

4. Why do you think that experiencing the Holocaust grants us the freedom to act as we wish, without having to consider the consequences on other peoples, or the possible response of our allies?

5. Why do you sympathize with people who think that in practice all world’s nations (including our allies) are against us and are striving for our annihilation (and that even some would say that from the days of "Amalek" to the present)?

6. Why do you sympathize with people who say that the Gaza Strip should be made into a “parking lot” whenever the Gaza people dare to make a sound?

7. Why do you think that the saying that the Israeli army is the moral army in the world is absolute, that cannot be doubted (an axiom), and as a consequence all IDF’s (Israeli Defense Forces) are moral by definition?

8. Why do you sympathize with those who think that the Palestinians are morally inferiors to all other world’s peoples? And that some would even say that their morality is similar to the morality of animals?

9. Why do you sympathize with people who believe that Israel always tries to promote peace, even during military operations, wars, and even when we decide to expand the construction of settlements in the West Bank?

10. Why do you think that the Palestinians use the peace talks with us as a mean to disguise they true aspiration – their plot to drive out all Jews to the sea?

1. Inconsistent (identity-supporting) interview

A short while ago you were asked to answer a short questionnaire that examined attitudes regarding the Israeli-Palestinian conflict. The aim of this part of the experiment is to examine these attitudes more deeply with a short interview. I ask you to think thoroughly about the questions I am about to ask you, and also to provide the reasoning for your answers. Let's begin:

1. In your opinion, what is the reason for that the Palestinians are the main victims in the course of the conflict?

2. Why do think that us, the Jewish people, are less moral, or at least, as moral as the Palestinian people?

3. Why do you think the real goal of the Palestinians is ultimately to live with us in peace?

4. Why do you think that our experience of the Holocaust should make us act morally toward other peoples, including the Palestinians?

5. Why do you sympathize with people that think that most countries in the world, ultimately, want the best for us, even when it seems that they criticize us, or make decisions that we do not like?

6. Why do you shy away from people who say that the Gaza Strip should be made into a “parking lot”?

7. Why do you think that the IDF is an army that can often act immorally?

8. Why do you think that the Palestinians are at least as moral as the rest of the world’s peoples?

9. Why do you sympathize with people that believe that Israel is doing everything in order to avoid peace promotion, including military operations, wars, and settlements expansion?

10. Why do you think that the Palestinians have one main goal when they approach peace talks, and that is to sign a peace treaty, that will allow them to live peacefully with us?

**A sample of auditory narratives used in the experiment in a given session before/after the intervention (translated from Hebrew)**

1. Incongruent political

1. Without further consideration, all the media define the Palestinians who were killed or injured at the checkpoints with the generic term "terrorists" - even before trying to find out if there was even a knife.

2. Israel left Gaza and since then claims that all the problems there are not its responsibility. But Israel actually contributed to the fact that Gaza has become a human prison where most of the residents live in poverty, and many children sometimes have to look for food in garbage cans.

3. Instead of ignoring repeated demands from most countries of the world, including the USA, Israel must stop building and expanding settlements in the territories of the Palestinian Authority.

4. In the past the British occupied this land and therefore the Jewish organizations rebelled against the British occupation. The same thing is happening now, when the Palestinians are rebelling against the Israeli occupation.

5. For many years Israel has been violating international and moral laws, including the most basic human rights of the Palestinians.

6. Sometimes the resistance of the Palestinians is violent, but this is natural - as other occupied nations have done throughout history.

1. Congruent political

1. The Palestinians have been repeatedly offered plausible ways to achieve statehood and autonomy. Instead of seizing one of these opportunities, they chose violence and a corrupt leadership that consists of murderers and terrorists.

2. In exchange for Israel's offer at Camp David in 2000, what it received in return was Palestinian terrorism, suicide terrorists and rocket launches towards the south of the country.

3. The restriction of the movement and freedom of the Palestinians in the areas under Palestinian authority are the result of the Palestinian terrorism which necessitates the military measures of Israel.

4. The situation will not improve until the Palestinians stop inciting terrorism and hatred, and accept the right of Israelis to live freely in their country. Israel has already accepted the two-state solution in the past; it is the Palestinians who did not accept it.

5. The daily terror experienced by Israelis mainly in East Jerusalem and Judea and Samaria only proves the need to protect these residents by any necessary means.

6. Sending suicide terrorists into Israel to kill civilians led to the establishment of the security wall. Therefore, it is the Palestinians who have to bear the responsibility for the difficulties in their lives that arise from the security wall.

1. Incongruent non-political

1. In recent years, Ritalin is hardly used as a drug to treat ADHD, and we have reached a situation where very few children in Israel take Ritalin.

2. Oil is a natural resource that will never run out. There will always be enough oil in the world, so in the future there will be no need to replace the fuel with alternative energy sources to drive cars or planes.

3. The technological development of the past hundreds of years does not stem from scientific progress, since science usually does not contribute to technological development. The main causes of technological development are astrological changes and God's will.

4. The claim that there is a sharp increase in the average temperature of the earth's surface during the last hundred years must be doubted, since those who claim so are scientists who falsified temperature data.

5. The frequency of flights for vacations abroad decreases significantly during holidays because the people of Israel enjoy being on vacation less during holidays and prefer to do so during the rest of the year.

6. Disposable plastic bags do not pollute the environment. In addition, their use is so rare that there is nothing to worry about and you can continue to use them without any restrictions or supervision.

1. Congruent non-political

1. Watermelon is a healthy and ideal fruit to eat especially in the hot summer months because it is sweet and satisfying and therefore protects against dehydration; It is also full of antioxidants and vitamins that are very nourishing to the body.

2. The Internet is a global computing network that connects billions of computers all over the world. In the last twenty years, the development of the Internet has gained momentum and today the Internet has become a major player in most areas of our lives.

3. Coral reefs are essential to the ecosystem in the oceans because they provide food and shelter for a large part of sea creatures. That is why care must be taken to protect the reefs that are still left in the world.

4. Wind turbines generate electricity from wind energy and thereby help reduce air pollution and greenhouse gases; Therefore, their use to generate electricity has been adopted in some countries of the world.

5. Environmental pollution often occurs from the extensive global use of oil and coal. Therefore, a shift to cleaner fuels or a more extensive use of public transportation should be encouraged.

6. Following the growth of population and industry in large cities, the air pollution in some of the largest cities in the world, such as Beijing and New Delhi, has become an almost unbearable environmental hazard that has long since crossed the levels considered safe for health.

**Results**

**The effect of the intervention on self-reported attitudes relating to the narratives.** To address the pre-registered hypotheses that the intervention, not the control manipulation, would moderate the political attitudes at the self-reported level, we examined the self-reported rating of perceived *Truthness* of the narratives and the *Affect* that they aroused – before and after the intervention. A mixed-design of repeated-measures between-subject ANOVA was conducted on these variables (*Truthness* and *Affect*) for each of the four different narratives conditions *(Political congruent, Political incongruent, non-Political congruent, non-Political incongruent*) for both experimental groups (Intervention and Control). First, as expected, and in-line with the pilot (c.f., SI), *Political incongruent* narratives were rated low on *Truthness* at T1 (2.21 ± .78 on a scale from 1 to 5) and T4 (2.23 ± .78 on a scale from 1 to 5), with a non-significant interaction for intervention (*F*(1, 72) = .39, *p* = .53, Eta^2^ = .005) or other main-effect (*F*(1, 72) < .07, *p* > .79, Eta^2^ < .002). They were also rated as evoking a medium level of *Affect* at T1 (3.19 ± .76 on a scale from 1 to 5) and T4 (3.19 ± .87 on a scale from 1 to 5), with a non-significant interaction for intervention (*F*(1, 72) = .82, *p* = .36, Eta^2^ = .01) or other main-effect (*F*(1, 72) < .04, *p* > .83, Eta^2^ < .002). Second, as expected and in-line with the pilot (c.f., Methods), *Political congruent* narratives were rated high on *Truthness* at T1 (4.19 ± .47 on a scale from 1 to 5) and at T4 (4.21 ± .54 on a scale from 1 to 5), with a non-significant interaction for intervention (*F*(1, 72) = 2.53, *p* = .12, Eta^2^ = .034) or other main-effect (*F*(1, 72) < .19, *p* > .66, Eta^2^ < .004). They were also rated as evoking a medium level of *Affect* at T1 (3.25 ± .80 on a scale from 1 to 5) and T4 (3.24 ± .84 on a scale from 1 to 5), with a non-significant interaction for intervention (*F*(1, 72) = .32, *p* = .57, Eta^2^ = .004) or other main-effect (*F*(1, 72) < .07, *p* > .79, Eta^2^ < .005). Third, as expected and in-line with the pilot (c.f., Methods), non-*Political congruent* narratives were rated high on *Truthness* at T1 (4.50 ± .40 on a scale from 1 to 5) and at T4 (4.45 ± .41 on a scale from 1 to 5) and evoking a low level of *Affect* at T1 (2.31 ± .61 on a scale from 1 to 5) and T4 (2.21 ± .73 on a scale from 1 to 5), while non-*Political incongruent* narratives were rated low on *Truthness* at T1 (1.67 ± .57 on a scale from 1 to 5) and at T4 (1.55 ± .56 on a scale from 1 to 5) and evoking a low level of *Affect* at T1 (2.29 ± .69 on a scale from 1 to 5) and T4 (2.32 ± .75 on a scale from 1 to 5). Intervention did not yield any significant effect on these non-Political narratives (*F*(1, 72) < .81, *p* > .37, Eta^2^ < .02).

In addition to examining the self-reported ratings of the narratives, we also examined the following variables before and after the intervention: *Ethos*, *Openness* and *Moral conviction*. We also examined two important variables after the intervention: *Unfreezing* and *Support for peace-promoting policies* (Hameiri, Porat, et al. 2014; Hameiri et al. 2016). First, a mixed-design of repeated-measures between-subject ANOVA was conducted on these three constructs and revealed the following: For *Ethos* there was a non-significant interaction (*F*(1, 73) = .17, *p* = .68, Eta^2^ = .002) but a main-effect (*F*(1, 73) = 4.92, *p* = .03, Eta^2^ = .063) such that *Ethos* was significantly reduced at T4 (4.48 ± .75 / 6) compared to T1 (4.59 ± .74 / 6). However, post-hoc analyses revealed that neither in the paradoxical intervention (*t*(33) = 1.60, *p* = .12) nor in the control group (*t*(40) = 1.50, *p* = .14) was there any significant difference in *Ethos* between T1 and T4. For *Openness* there was a non-significant interaction (*F*(1, 73) = 2.40, *p* = .13, Eta^2^ = .03) or main-effects (*F*(1, 73) < .20, *p* > .65, Eta^2^ < .004). For *Moral conviction* there was a non-significant interaction (*F*(1, 73) = .007, *p* = .93, Eta^2^ < .001) or main-effects (*F*(1, 73) < 3.24, *p* > .07, Eta^2^ < .05). Finally, *Unfreezing* was not significantly (*t*(76) = .86, *p* = .39) different in the intervention (17.02 ± 17.54 / 100) compared to the control (20.49 ± 18.05 / 100) group, nor was *Support for peace-promoting policies* significantly (*t*(76) = 1.00, *p* = .32) different in the intervention (3.45 ± .96 / 6) compared to the control (3.64 ±.73 / 6) group.

**Source reconstruction projections of multiple MCCA statistical contrasts.**


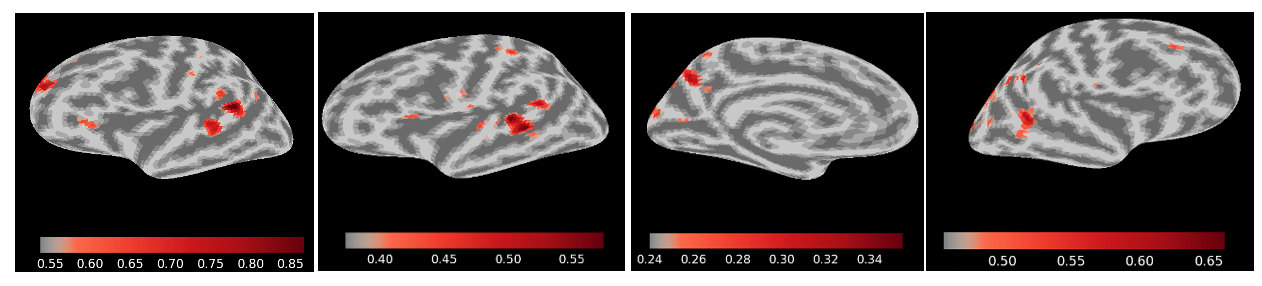


**Figure S1**. *Cortical source reconstruction projections for the T3 vs T2 MCCA contrasts for the following group conditions (from left to right): intervention incongruent, intervention congruent, control incongruent and control congruent. Colorbars convey activation strength of mcca.*


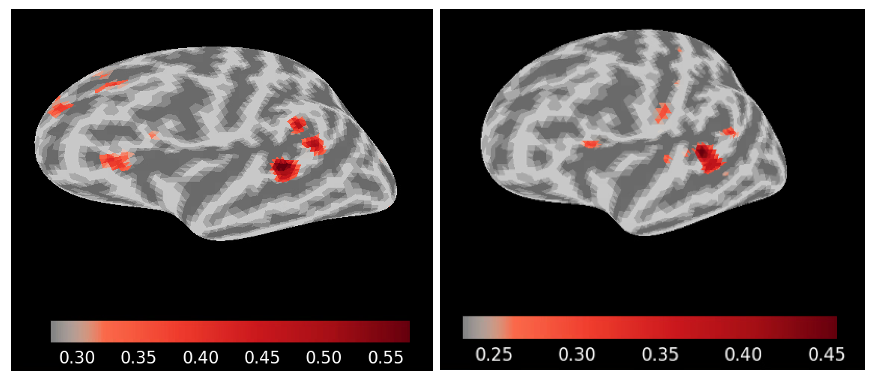


**Figure S2**. *Cortical source reconstruction projections for the double MCCA contrasts: the interventional contrasts (T3 vs T2) which are contrasted for the political vs non-political conditions: for the incongruent (left panel) and congruent (right panel) data. Colorbars convey activation strength of mcca.*
